# Influence of Supplementation of Lactoferrin, Melittin and Cecropin A to Rat Diet on Changes in Faecal Ammonia Concentrations, Short-Chain Fatty Acid Concentrations and Activities of Bacterial Enzymes

**DOI:** 10.3390/ani11051203

**Published:** 2021-04-22

**Authors:** Jerzy Juśkiewicz, Aleksandra Rawicka, Bartosz Fotschki, Michał Majewski, Zenon Zduńczyk

**Affiliations:** 1 Institute of Animal Reproduction and Food Research, Department of Biological Function of Food, Polish Academy of Sciences, 10-748 Olsztyn, Poland; b.fotschki@pan.olsztyn.pl (B.F.); z.zdunczyk@pan.olsztyn.pl (Z.Z.); 2 Policlinico Veterinario Roma Sud, Ophthalmology, 00173 Rome, Italy; o.wiczkowska@gmail.com; 3 Department of Pharmacology and Toxicology, Faculty of Medicine, University of Warmia and Mazury, 10-082 Olsztyn, Poland; michal.majewski@uwm.edu.pl

**Keywords:** antimicrobial peptides, melittin, cecropin A, lactoferrin, faeces, bacterial enzymes, short-chain fatty acids, ammonia, rat

## Abstract

**Simple Summary:**

In the present study conducted on Wistar laboratory rats, the effects of two selected insect antimicrobial peptides (AMPs), melittin and cecropin A, were investigated and compared to those attributed to well-known antibacterial action of lactoferrin. It was hypothesised that the dietary presence of lactoferrin, melittin or cecropin A strongly affects the rat large gut microbial activity at the time of protein/peptide administration and the durability of the effects may differ after their withdrawal from a diet. The experiment was conducted on living animals (without their euthanasia) and the dynamics of changes in pH, microbial enzyme activity, ammonia and short-chain fatty acids concentrations were investigated in the faeces during and after the dietary treatments with lactoferrin, melittin or cecropin A. The results suggested that the faecal intensity of microbial fermentation processes in rats was quickly reduced upon dietary addition of two AMPs and lactoferrin after two days of treatment, on average. The strongest suppression effect was observed on the 5th day of treatment and persisted on days 5–8. The changes caused by the supplemented lactoferrin and AMPs were reversible after 15 days, i.e., 10 days after the withdrawal of lactoferrin, melittin and cecropin A from the diet.

**Abstract:**

We hypothesised that the dietary addition of the bioactive antimicrobial protein lactoferrin (LF) and peptides melittin (MT) or cecropin A (CR) at a dosage of 100 mg/kg to the diet of Wistar rats would result in strong modulatory effects on faecal microbial enzymatic activity, short-chain fatty acid and ammonia concentrations. To date, the changes in bacterial extracellular and intracellular enzymatic activities upon addition of dietary AMPs have not yet been studied. This experiment lasted 15 days; during the first 5 day period, the rats were fed the control diet (S) and diets supplemented with LF, MT or CR. On days 6–15, all rats were fed the control S diet. The faecal fermentation processes were substantially stopped after two days of treatment, on average, in all rats receiving LF and two AMPs. The deepest suppression effect was observed on the last day of treatment (day 5) and persisted through days 5–8. The highest decreases in faecal bacterial β-glucosidase and β-glucuronidase activities as well as in SCFA and ammonia concentrations were observed in the rats fed the CR diet. Only in the CR animals did the mechanism of suppressed microbial fermentation involve diminished enzyme release from bacterial cells to the digesta.

## 1. Introduction

Antimicrobial peptides (AMPs) are natural components of innate immunity in plants, fungi, bacteria and animals [1]. These small molecules (generally 2–50 kDa) serve as potent tools for controlling the number and activity of pathogens and natural microorganisms. AMPs are rapidly produced by the host in exposed tissues (skin, lungs, intestine, blood) at the time of contact with pathogens, but they are also stored as an emergency supply in cells [2]. It has been reported that insects, as organisms strongly resistant to invading bacteria, are able to produce massive amounts of AMPs at the time of danger [3]. Insect AMPs are classified into three types: α-helical, β-sheet cysteine-rich and linear-extended peptides. Cecropins and cecropin-like peptides form the most abundant family of α-helical AMPs with relatively high in vitro antimicrobial activity against Gram-negative bacteria and, to a lesser extent, against Gram-positive bacteria [4]. Interestingly, cecropins have been supposed to exhibit weak toxicity against normal mammalian cells and almost no effect on haemolysis in erythrocytes [5]. cecropin A, one of the well-researched cecropins, was first discovered in Hyalophora cecropia and has been used as a template for peptide molecular hybrids such as melittin [6]. Despite in vitro-derived knowledge, little is known about the in vivo effects of cecropin A on gut microbiota [4]. Regarding melittin, a polypeptide that constitutes half of dry honeybee venom, although much is known about its strong interaction with lipid membranes leading to increased permeability of erythrocytes and other cell membranes, less is known about how it affects the metabolism of the intestinal microbiota [7]. It has been predicted that these small peptide molecules may destroy bacterial cells by membrane and cytoplasmic component disruption and/or by interference with some metabolic pathways [8]. For instance, AMPs can demolish intracellular enzymes and DNA when entering the pathogen [9]. The relationship between ingested antibacterial agents and the gut microbiome is typically characterised by a double-face effect, with possible adverse effects from one side and with possible beneficial application from the other side. In our recent study [10], an analysis of bacterial enzymatic activity in rat caecal digesta revealed that lower short-chain fatty acid (SCFA) concentrations in the presence of antibacterial nanoparticles could be attributed to significantly diminished bacterial total enzymatic activity, including both extracellular and intracellular activities. The extracellular activity of bacterial enzymes has direct implications for the rate at which nutrients and non-nutrients undergo microbial digestion in the large intestine. Extracellular enzyme activity is influenced by the type and number of bacterial species present in the intestinal/faecal digesta and by the rate of enzyme secretion by bacterial cells. Gugolek et al. [11] reported that an elevated extracellular activity of glycoside hydrolases in relation to the total (sum of extra- and intracellular) activity of those enzymes could be an important adaptive mechanism of bacteria living in unfavourable intestinal environments. To the best of our knowledge, the changes in bacterial extracellular and intracellular enzymatic activities upon dietary AMPs have not yet been extensively studied. Taking that into account, the effects of two selected insect AMPs, melittin and cecropin A, were investigated and compared to those attributed to the well-known antibacterial action of lactoferrin. Lactoferrin is present in higher quantities in saliva, tears, seminal fluid, white blood cells and the milk of mammals [12]. It has been revealed that lactoferrin has bacteriostatic properties against Gram-negative bacteria requiring high iron, such as coliforms [13].

In the present study, it was hypothesised that the dietary presence of the antimicrobial protein—lactoferrin—and antimicrobial peptides—melittin or cecropin A—would strongly affect rat large gut microbial activity at the time of their administration and that the durability of the effects may differ after their withdrawal from a diet. The main intestinal segment of microbial fermentation processes in rats is the caecum. The measurements of caecal parameters would involve animal euthanasia, therefore with respect to 3Rs concept (replacement, reduction and refinement), the dynamics of changes in pH, microbial enzyme activity, ammonia and short-chain fatty acid concentrations were investigated in the faeces during and after dietary treatments with lactoferrin and the antimicrobial peptides (melittin or cecropin A).

## 2. Materials and Methods

### 2.1. Antimicrobial Peptides and Lactoferrin

Lactoferrin, melittin and cecropin A were purchased from Sigma-Aldrich Co. (Poznań, Poland). The purity of the preparations (according to the manufacturer) was L4765 Lactoferrin ≥ 85% (SDS-PAGE), M2272 Melittin ≥ 85% (HPLC) and C6830 cecropin A ≥ 97% (HPLC).

### 2.2. In Vivo Experiment

The in vivo experiment was conducted on 32 adult, male, Wistar outbred rats (*Rattus norvegicus*, Cmdb:WI). The animals were used in compliance with the European Guidelines for the Care and Use of Laboratory Animals [14]. The experimental protocol was permitted by the Local Animal Care and Use Committee (Approval No. 34/2019; Olsztyn, Poland). All efforts were made to minimise experimental animal suffering. Two weeks before the start of the experiment, the rats were fed a standard non-supplemented diet prepared on a laboratory scale based on the recommendation of AIN-1993 [15]. The diet contained 20% casein (Lacpol Co., Murowana Goślina, Poland), 0.3% DL-methionine (SIGMA, Poznań, Poland), 0.3% cholesterol, 0.2% choline chloride, 8% rapeseed oil, 8% cellulose, standard mineral and vitamin mixes (3.5 and 1%, respectively; according to AIN-93 requirements of laboratory adult rats), and corn starch to total 100%. That diet was also used as a control dietary treatment (S group; Table 1).

The rats were similar in body weight (BW) values (285 ± 11.6 g) and were kept individually in metabolic cages (Tecnipalst, Buguggiate, Italy), enabling faecal sample collection. The feeding experiment lasted 15 days and comprised two periods: 5 days and 10 days. During the first 5 day period, the rats were subjected to four dietary treatments, i.e., control (S) without any supplementation; LF, supplemented with lactoferrin (100 mg/kg); MT, supplemented with melittin (100 mg/kg); and CR, supplemented with cecropin A (100 mg/kg). To evenly blend the peptide preparation with other diet components, the peptide was added along with rapeseed oil (Table 1). A fresh diet was served daily ad libitum, and access to water was continuous. In the second 10-day period, all rats were treated with the control diet. Standard conditions at 21–22 °C and relative air humidity of 50% with intensive room ventilation (15 × per h) and a 12 h light/12 h dark regimen was applied. During the entire feeding period, from 6.00 p.m.–9.00 p.m. on days 0, 1, 2, 5, 6, 8, 10 and 15, rat faeces were thoroughly collected and subjected to the analyses as soon as possible. The daily consumed dosage of the LF, MT and CR per rat was 7 mg/kg of BW, and it reflected a daily ingestion of 85 mg of the supplemented protein/peptide by an adult weighing 75 kg (calculated with the aid of the body surface area method [16]).

### 2.3. Measurements and Analyses

Samples of fresh faeces were combined in a porcelain laboratory mortar and then used for the following analyses: pH, bacterial enzymatic activity and concentrations of ammonia and short-chain fatty acids (SCFAs). The faecal pH was measured immediately (three measurements per sample) using a microelectrode and a pH/ION meter (model 301, Hanna Instruments, Vila do Conde, Portugal). Ammonia was extracted from fresh faeces, trapped in a solution of boric acid in Conway dishes and determined by direct titration with sulfuric acid [17]. The concentrations of SCFAs in the samples were analysed by gas chromatography (Shimadzu GC-2010, Kyoto, Japan). The samples (0.2 g) were mixed with 0.2 mL of formic acid, diluted with deionised water and centrifuged at 7211× *g* for 10 min. The supernatant was transferred to a vial and loaded onto a capillary column (SGE BP21, 30 m × 0.53 mm) using an on-column injector. The initial oven temperature was 85 °C, and it was raised to 180 °C in steps of 8 °C/min and maintained for 3 min. The temperatures of the flame ionisation detector and the injection port were 180 °C and 85 °C, respectively. The volume of the sample for gas chromatography was 1 µL. The concentrations of faecal putrefactive SCFAs (PSCFAs) were calculated as the sum of isobutyrate, isovalerate and valerate. All SCFA analyses were performed in duplicate. Pure acetic, propionic, butyric, iso-butyric, iso-valeric and valeric acids were obtained from Sigma (Poznan, Poland), and they were combined to create a standard plot and calculate the amount of each acid. The additional set of pure acids was included in each GC run at five sampling intervals to maintain calibration.

In addition to SCFA analysis, faecal fermentation processes were analysed based on the activity of selected bacterial enzymes (α- and β-glucosidase, β-glucuronidase), which was measured by the rate of release of p-nitrophenol from the respective nitrophenylglucosides, according to a previously described method [18]. The following substrates were used: p-nitrophenyl-α-D-glucopyranoside (for α-glucosidase), p-nitrophenyl-β-D-glucopyranoside (for β-glucosidase) and p-nitrophenyl-β-D-glucuronide (for β-glucuronidase). The activity of enzymes secreted by bacterial cells in the faecal environment was measured by preparing a reaction mixture containing 0.3 mL of the substrate solution (5 mM) and 0.2 mL of a 1:10 (*v/v*) dilution of the faecal sample in 100 mM phosphate buffer (pH 7.0), which was centrifuged at 7211× *g* for 15 min. Incubation was carried out at 37 °C, and p-nitrophenol was quantified at 400 nm after the addition of 2.5 mL of 0.25 M cold sodium carbonate. Enzyme activity was expressed in μmol of the product formed per hour per gram of fresh faeces. To determine the total activity of selected faecal bacterial enzymes, including extracellular activity (see the procedure above) and intracellular activity, a sample of faeces diluted in phosphate buffer was mechanically disrupted by vortexing with glass beads (212–300 μm in diameter; four periods of 1 min each, with 1 min cooling intervals on ice) in the FastPrep^®^-24 homogenizer (MP Biomedicals, Santa Ana, CA, USA). The resulting mixture was centrifuged at 7211 *g* for 15 min at 4 °C. The supernatant was used for the enzyme assay described above. Intracellular enzyme activity was calculated by comparing total enzyme activity with the activity of bacterial enzymes released into the faecal environment, and it was expressed in μmol of the product (PNP, p-nitrophenol) formed per hour per gram of faeces. The respective calculation formulas were derived based on the model curves for PNP (PNP standard solution in 100 mM phosphate buffer, pH 7.0, 40 mg/L). Extracellular enzyme activity was also determined as the rate of enzyme release, and it was expressed as a percentage of total enzyme activity. All analyses were performed in duplicate.

### 2.4. Statistical Analyses

Data are expressed as the mean and pooled standard error of the mean (SEM). The results were analysed statistically by one-way ANOVA, and the significance of differences between groups was determined with the Tukey multiple range test at a significance level of *p* ≤ 0.05. All calculations were performed with the aid of Statistica 12.0 software.

## 3. Results

### 3.1. Faecal β-Glucosidase Activity

**Figure 1:**
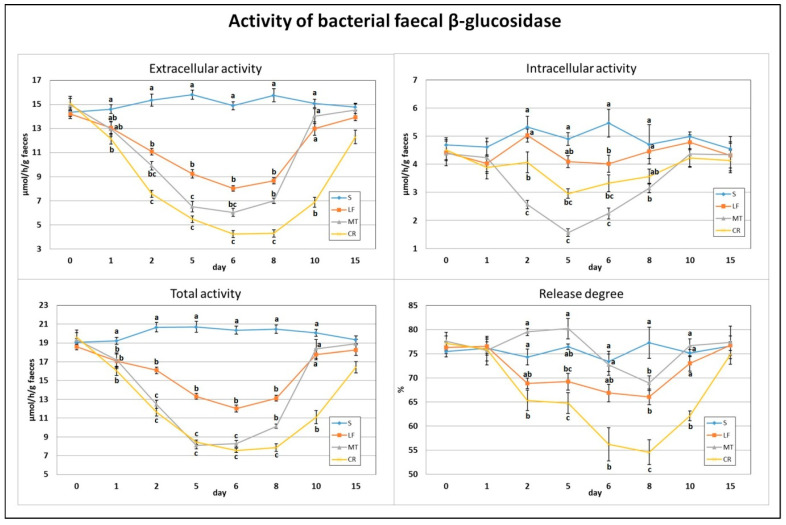
Experimental factor in particular groups: S—control standard diet (0–15 days); LF—standard diet with lactoferrin addition 100 mg/kg (0–5 days diet LF and 6–15 days diet S); MT—standard diet with melittin addition 100 mg/kg (0–5 days diet MT and 6–15 days diet S); CR—standard diet with cecropin A addition 100 mg/kg (0–5 days diet CR and 6–15 days diet S). ^a,b,c^ Mean values on the day with no common superscript are different at *p* ≤ 0.05. Data are presented as the mean with SEM; SEM—SD for all rats divided by the square root of rat number in one group (*n* = 8). Release degree, extracellular expressed as percent of total activity.

After just one day of the treatment, the extracellular activity of faecal bacterial β-glucosidase was decreased by the CR diet compared to the control diet (Figure 1). The total activity, composed of extra- and intracellular activities, of that enzyme on day 1 was decreased by LF and two AMP treatments (*p* < 0.05 vs. S). The highest and lowest extracellular activity on day 2 was noted in the S (*p* < 0.05 vs. remaining groups) and CR (*p* < 0.05 vs. S, LF) animals, respectively. On that day, the highest and lowest intracellular activity was in rats in the S, LF and MT groups (*p* < 0.05 vs. MT, CR and *p* < 0.05 vs. S, LF, CR, respectively). The total activity of faecal bacterial β-glucosidase on day 2 was significantly reduced by LF and two AMPs compared to the control, but the strongest reduction was noted in the MT and CR rats (*p* < 0.05 vs. S and LF). On day 2, the calculated release of enzymes was significantly diminished by the CR treatment in comparison to the S and MT treatments. After 5 days of feeding with diets containing LF and AMPs, the faeces of rats in the MT and CR groups were characterised by the lowest extracellular and total β-glucosidase activities (*p* < 0.05 vs. S and LF), while the highest activities were attributed to the S group (*p* < 0.05 vs. all remaining groups). For the intracellular activity on day 5, the lowest value was in MT rats (*p* < 0.05 vs. S, LF), and the highest was in S rats (*p* < 0.05 vs. MT, CR). The percentage of enzyme release on day 5 was the highest and lowest in the MT and CR groups, respectively (*p* < 0.05 vs. LF, CR and *p* < 0.05 vs. S, MT). On day 6, the S animals had the highest faecal total, extra- and intracellular activities of bacterial β-glucosidase (*p* < 0.05 vs. other groups); the lowest total activity was in the MT and CR rats, the lowest extracellular activity was in the CR group, and the lowest intracellular activity was in the MT group (in all cases *p* < 0.05 vs. S and LF). The enzyme release value on day 6 was decreased by CR treatment compared to the S and MT treatments. The highest and lowest values for extracellular activity, total activity and enzyme release of β-glucosidase on day 8 were noted in the S and CR faeces (in all cases *p* < 0.05 vs. other groups). On day 8, the intracellular activity was diminished by the MT treatment (*p* < 0.05 vs. S and LF). The faeces of CR rats were characterised by decreased extracellular activity, total activity and enzyme release percentage on day 10 (*p* < 0.05 vs. S, LF and MT). On day 15, the differences in assessed β-glucosidase indicators between groups were insignificant (*p* > 0.05).

### 3.2. Faecal β-Glucuronidase Activity

**Figure 2:**
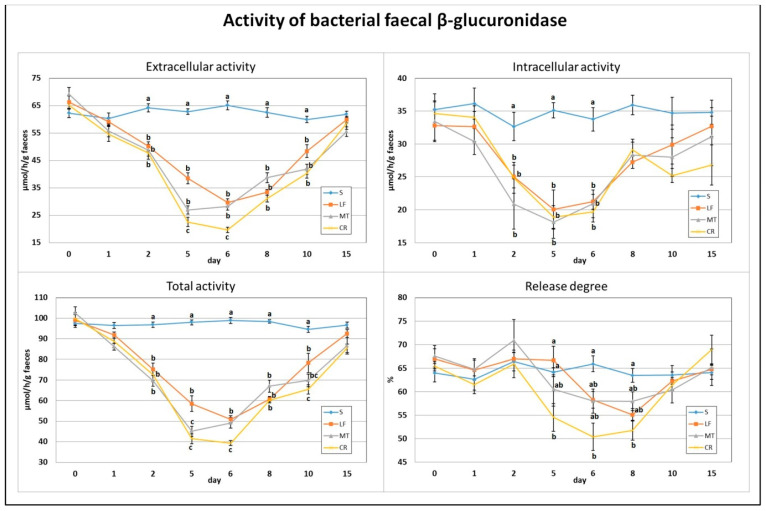
Experimental factor in particular groups: S—control standard diet (0–15 days); LF—standard diet with lactoferrin addition 100 mg/kg (0–5 days diet LF and 6–15 days diet S); MT—standard diet with melittin addition 100 mg/kg (0–5 days diet MT and 6–15 days diet S); CR—standard diet with cecropin A addition 100 mg/kg (0–5 days diet CR and 6–15 days diet S). ^a,b,c^ Mean values on the day with no common superscript are different at *p* ≤ 0.05. Data are presented as the mean with SEM; SEM—SD for all rats divided by the square root of rat number in one group (*n* = 8). Release degree, extracellular expressed as percent of total activity.

On day 2, a significant decrease in the total, extracellular and intracellular activities of bacterial faecal β-glucuronidase was found for all three dietary AMP treatments compared to the S group (Figure 2). Similar decreases in the LF, MT and CR rats were observed on days 5 and 6. Additionally, on day 5, the MT and CR dietary treatments and, on day 6, only the CR treatment were accompanied by a higher drop in the total and extracellular activities (*p* < 0.05 vs. S and LF and *p* < 0.05 vs. S, LF and MT, respectively). The CR group had a lower faecal β-glucuronidase release degree on days 5 and 6 (*p* < 0.05 vs. S, LF and *p* < 0.05 vs. S, respectively). On days 8 and 10, all three AMP treatments caused diminished total and extracellular activity in comparison to the S treatment (*p* < 0.05). Additionally, on day 10, the CR treatment was associated with the lowest faecal total activity of bacterial β-glucuronidase (*p* < 0.05 vs. S and LF). The percentage of enzyme release on day 8 was the lowest in the CR faeces (*p* < 0.05 vs. S).

### 3.3. Faecal α-Glucosidase Activity

**Figure 3:**
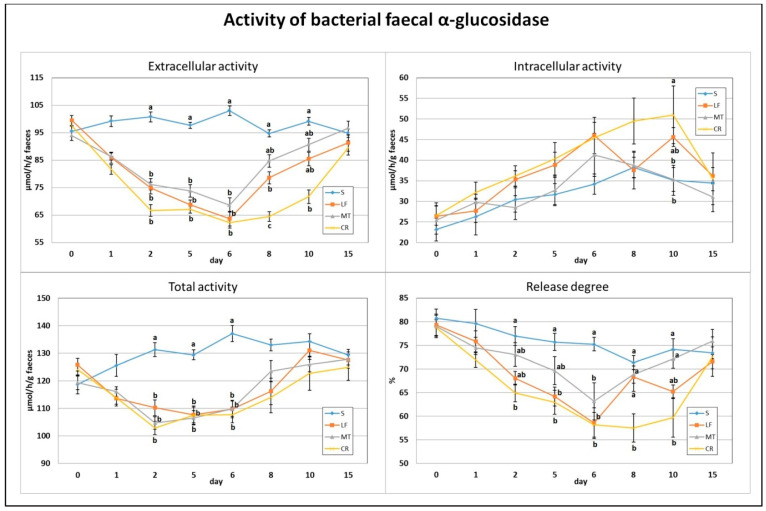
Experimental factor in particular groups: S—control standard diet (0–15 days); LF—standard diet with lactoferrin addition 100 mg/kg (0–5 days diet LF and 6–15 days diet S); MT—standard diet with melittin addition 100 mg/kg (0–5 days diet MT and 6–15 days diet S); CR—standard diet with cecropin A addition 100 mg/kg (0–5 days diet CR and 6–15 days diet S). ^a,b,c^ Mean values on the day with no common superscript are different at *p* ≤ 0.05. Data are presented as the mean with SEM; SEM—SD for all rats divided by the square root of rat number in one group (*n* = 8). Release degree, extracellular expressed as percent of total activity.

The applied dietary addition of three AMPs, lactoferrin, melittin and cecropin A, resulted in a significant decrease in faecal total and extracellular activity of bacterial α-glucosidase on days 2, 5 and 6 (*p* < 0.05 vs. S; Figure 3). In comparison to the S treatment, the calculated enzyme release percentage was diminished on day 2 in the CR treatment, on day 5 in the CR and LF treatments, and on day 6 in the CR, LF and MT treatments (*p* < 0.05). The lowest extracellular activity of faecal α-glucosidase and its enzyme release percentage on day 8 were noted in the CR group (in both cases *p* < 0.05 vs. all remaining groups). Additionally, on that day, the LF rats had significantly lower extracellular activity than the S rats fed the control diet. On day 10, the CR group had the lowest extracellular and the highest intracellular activities of faecal α-glucosidase (*p* < 0.05 vs. S and *p* < 0.05 S and MT, respectively). The percentage of enzyme release on day 10 was significantly decreased by the CR treatment in comparison to the S and MT treatments.

### 3.4. Faecal SCFA Concentration

**Figure 4:**
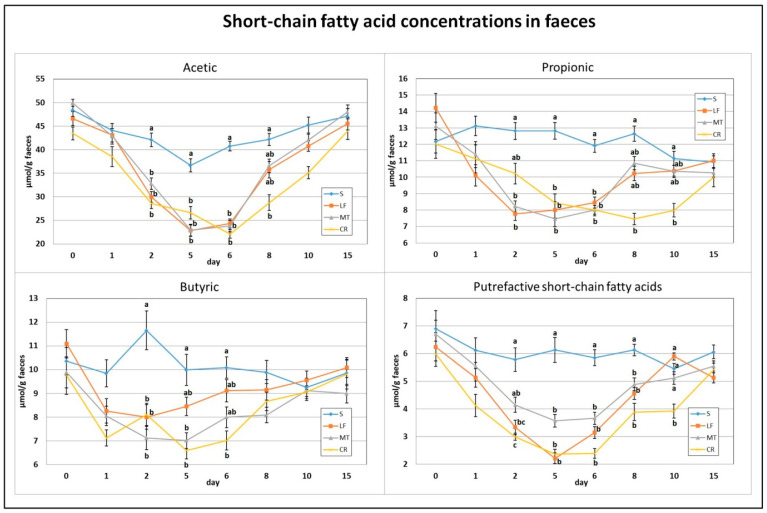
Experimental factor in particular groups: S—control standard diet (0–15 days); LF—standard diet with lactoferrin addition 100 mg/kg (0–5 days diet LF and 6–15 days diet S); MT—standard diet with melittin addition 100 mg/kg (0–5 days diet MT and 6–15 days diet S); CR—standard diet with cecropin A addition 100 mg/kg (0–5 days diet CR and 6–15 days diet S). ^a,b,c^ Mean values on the day with no common superscript are different at *p* ≤ 0.05. Data are presented as the mean with SEM; SEM—SD for all rats divided by the square root of rat number in one group (*n* = 8).

**Figure 5:**
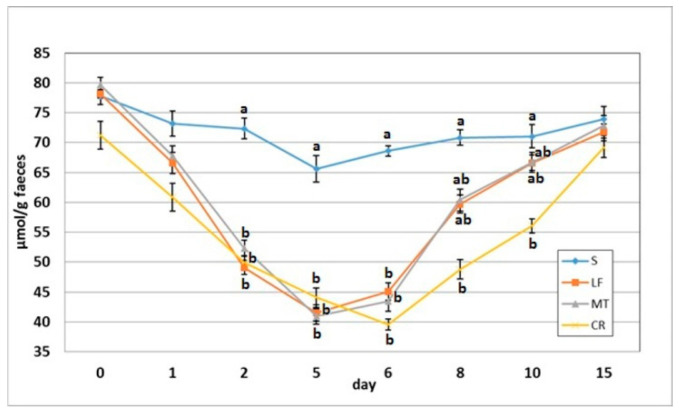
Total short-chain fatty acid concentration in faeces of rats fed diets supplemented with lactoferrin, melittin or cecropin A. Experimental factor in particular groups: S—control standard diet (0–15 days); LF—standard diet with lactoferrin addition 100 mg/kg (0–5 days diet LF and 6–15 days diet S); MT—standard diet with melittin addition 100 mg/kg (0–5 days diet MT and 6–15 days diet S); CR—standard diet with cecropin A addition 100 mg/kg (0–5 days diet CR and 6–15 days diet S). ^a,b^ Mean values on the day with no common superscript are different at *p* ≤ 0.05. Data are presented as the mean with SEM; SEM—SD for all rats divided by the square root of rat number in one group (*n* = 8).

As indicated in Figure 4 and Figure 5, on day 2, the dietary treatments supplemented with LF, MT and CR caused a significant decrease in the faecal concentrations of acetic acid, butyric acid and total SCFAs in comparison to the control treatment. The propionic acid concentration was decreased by treatments LF and MT, and the faecal PSCFA concentration was decreased by treatments LF and CR compared to the S treatment (*p* < 0.05). The lowest PSCFA level on day 2 was attributed to the CR treatment (*p* < 0.05 vs. all other groups). In comparison to the control group, the three supplemented AMP groups had decreased faecal concentrations for all analysed SCFAs on days 5 and 6, except for the butyric acid concentration in the faeces of the LF group and that in the faeces of the LF and MT groups, on those days respectively. On day 8, the three AMPs reduced the faecal concentration of PSCFAs, while the CR treatment decreased the total SCFA, acetic acid and propionic acid concentrations (*p* < 0.05 vs. S in all cases). The CR group on day 10 was characterised by the lowest faecal concentrations of propionic acid, total SCFAs (*p* < 0.05 vs. S) and faecal PSCFAs (*p* < 0.05 vs. S, LF, MT).

### 3.5. Faecal pH and Ammonia Concentration

**Figure 6:**
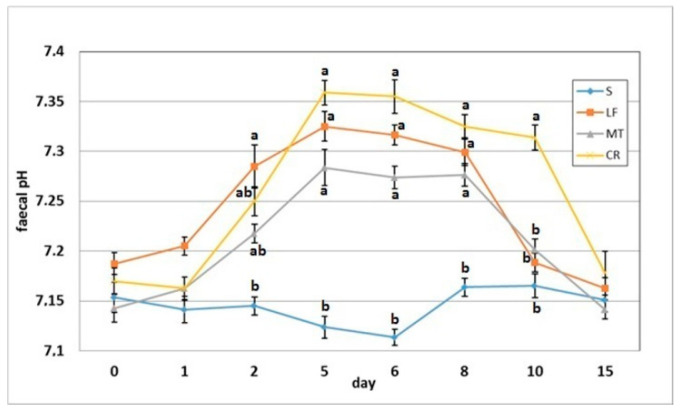
Faecal pH of rats fed diets supplemented with lactoferrin, melittin or cecropin A. Experimental factor in particular groups: S—control standard diet (0–15 days); LF—standard diet with lactoferrin addition 100 mg/kg (0–5 days diet LF and 6–15 days diet S); MT—standard diet with melittin addition 100 mg/kg (0–5 days diet MT and 6–15 days diet S); CR—standard diet with cecropin A addition 100 mg/kg (0–5 days diet CR and 6–15 days diet S). ^a,b^ Mean values on the day with no common superscript are different at *p* ≤ 0.05. Data are presented as the mean with SEM; SEM—SD for all rats divided by the square root of rat number in one group (*n* = 8).

**Figure 7:**
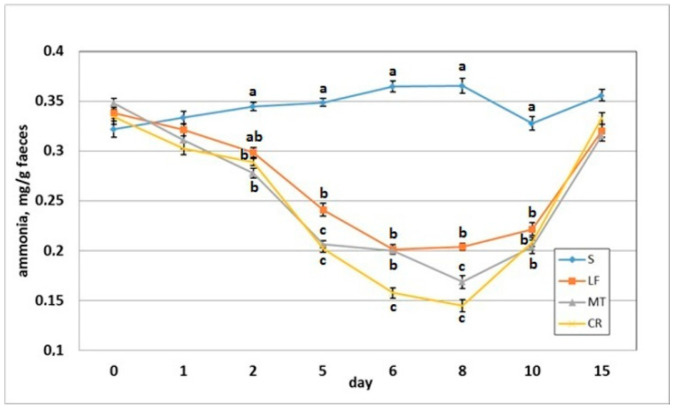
Faecal ammonia concentration of rats fed diets supplemented with lactoferrin, melittin or cecropin A. Experimental factor in particular groups: S—control standard diet (0–15 days); LF—standard diet with lactoferrin addition 100 mg/kg (0–5 days diet LF and 6–15 days diet S); MT—standard diet with melittin addition 100 mg/kg (0–5 days diet MT and 6–15 days diet S); CR—standard diet with cecropin A addition 100 mg/kg (0–5 days diet CR and 6–15 days diet S). ^a,b,c^ Mean values on the day with no common superscript are different at *p* ≤ 0.05. Data are presented as the mean with SEM; SEM—SD for all rats divided by the square root of rat number in one group (*n* = 8).

The dietary addition of LF resulted in a significant increase in the faecal pH on day 2 when compared to the S group (Figure 6). The AMP experimental treatments LF, MT and CR elevated the faecal pH on days 5, 6 and 8 compared with that of the control treatment (*p* < 0.05). The CR rats had increased faecal pH on day compared to the S, LF and MT animals (*p* < 0.05). All rats had similar faecal pH values on day 15 within a relatively narrow range of 7.14–7.18. The faecal ammonia concentration on day 2 was significantly decreased in rats fed diets MT and CR compared to those fed diet S (Figure 7). The MT and CR groups had the lowest faecal ammonia concentrations on days 5 and 8 (*p* < 0.05 vs. S and LF), while the highest concentration on those days was noted in the S group (*p* < 0.05 vs. remaining groups). The highest and lowest ammonia levels on day 6 were observed in the faeces of the S and CR animals, respectively (in both cases *p* < 0.05 vs. other groups). The three AMP treatments caused similar significant decreases on day 10 in the faecal ammonia concentration vs. the control treatment. On day 15, the differences in faecal ammonia between groups were insignificant (*p* > 0.05).

## 4. Discussion

In the present study, the faecal concentration of microbial fermentation end-products (short-chain fatty acids, ammonia) and the activity of selected bacterial enzymes were analysed instead of bacterial count. Of course, bacteriological investigations (e.g., identification of selected groups) should be regarded as useful in describing the basic ecology of the gut, but they do not describe and explain metabolic details that directly impact the health status of the host. Currently, modern research techniques allow us to conduct studies of the metabolome, i.e., the complete set of metabolites produced within an organism, but this is still an expensive and labour-intensive process [19]. An alternative approach is to use biochemical assays that measure the functional activity of the microbiota as a whole and thus permit deductions to be made regarding the role of the microbial community in the metabolism of dietary components. In addition, by selecting microbial enzyme activities or metabolic endpoints resulting in compounds with potentially toxic or beneficial effects, probable health consequences for the host can be assessed [20,21]. To better characterise the enzymatic action of the microbial community, in the present study, the extracellular, intracellular and total activities of bacterial enzymes were measured in the faecal environment. It has been reported that extracellular enzyme activity directly affects the rate at which nutrients and non-nutrients undergo microbial digestion in the lower GIT [22,23]. In turn, the total activity of enzymes composed of extra- and intracellular activities reflected the type and counts of bacterial species present in the digesta. In the present experiment, the modulatory effects of the dietary application of lactoferrin and the AMPs (melittin and cecropin A) on bacterial α-glucosidase, β-glucosidase and β-glucuronidase were thoroughly scrutinised for 15 days, of which the first five days were the period of dietary AMP addition. The total, extracellular and intracellular activities of these enzymes quickly decreased, on average, after two days of AMP application, while the highest activity drop was noted on the last day (day 5) of dietary peptide administration and lasted for the next two to three days. It has been reported that AMPs lower bacterial counts through a variety of mechanisms, including membrane disruption, interference with bacterial metabolism, and through targeting cytoplasmic components [1]. The reduced total activity of faecal bacterial enzymes upon application of AMPs should be ascribed to both lowered bacterial count and depressed metabolic activity of microbes in the faecal digesta. Interestingly, the diminished activity of α-glucosidase, β-glucosidase and β-glucuronidase caused by the dietary presence of cecropin A was additionally accompanied by a decreased rate of enzyme release from bacterial cells into the faecal environment. Such an effect was almost absent in the case of dietary supplementation with lactoferrin and melittin. It is well known that AMPs can change the permeability of the cell membrane, but different action mechanisms have been proposed, including barrel-stave, carpet, toroidal-pore and others [24]. The precise antibacterial action of cecropin A is still undiscovered, although recent work by Yun and Lee [25] showed that cecropin A-induced apoptotic activity was regulated by an ion imbalance and glutathione antioxidant system. Lee et al. [26] provided a new insight into the in vitro cecropin A mechanism of action with the aid of mouse macrophage-derived cells stimulated with lipopolysaccharides (LPS). Those authors observed that the antibacterial activity of cecropin A towards multidrug-resistant Gram-negative *Acinetobacter baumanii* and *Pseudomonas aeruginosa* was much higher than that of melittin. They also showed lower hemolytic activity and cytotoxicity of cecropin A compared to melittin, the latter in relation to their minimal inhibitory concentration (MIC). As opposed to melittin, cecropin A was found to be profoundly selective for the above-mentioned multidrug-resistant bacterial cells in the test with negatively charged and zwitterionic phospholipid vesicles, which mimic the Gram-negative bacterial and mammalian cell membranes, respectively [26]. Those differences between cecropin A and melittin might help explain the decreased rate of enzyme release from bacterial cells into the faecal environment in the CR rats observed in the present study. Additionally, a recent study conducted by Kalsy et al. [27] revealed a multi-target mechanism ascribed to antimicrobial peptide cecropin A entailing the pore-forming activity, inhibition of efflux pump activity and interactions with extracellular/intracellular nucleic acids. Wu et al. [1] reported that dietary supplementation with cecropin A beneficially alleviated enterotoxigenic Escherichia coli-induced piglet diarrhoea. Of paramount importance is the fact that Gram-negative bacteria have the presence of LPS on their cell surface, which are responsible for inflammatory reactions and septic shock in the host. In that regard, it has been observed that enhanced activity of bacterial β-glucuronidase followed the undesired growth of Escherichia coli and Clostridium intestinal populations [28]. β-Glucuronidase is involved in the generation of toxic and potentially harmful metabolites in the hindgut [29]. Regarding the accepted hypothesis, the CR treatment caused the highest drop in faecal β-glucuronidase activity, with the maximum drop noted on days 5 and 6. The decreased β-glucuronidase activity persisted until day 10 in all three AMP groups, but again, the CR faeces were characterised by their lowest total activity.

The substrates of bacterial glycolytic enzymes and the functional and health implications of their products have been extensively researched [30,31]. For example, α-glucosidase activities can improve fermentation of resistant starch, leading to the production of SCFAs and lactic acids, which are a source of energy for both the intestinal microbial community and the host tissues [32]. In contrast to glucoamylases (glucan 1,4-α-glucosidases), α-glucosidases favour oligosaccharides as substrates, while polysaccharides are hydrolysed relatively slowly or not at all. Numerous α-glucosidases from bacteria and eukaryotes have been characterised, and the majority of them have been from mesophilic organisms [33]. In the present study, the diminished activity of faecal α-glucosidase was comparable in the LF and MT groups. Similar to the case of bacterial β-glucuronidase, the strongest effect on α-glucosidase activity followed the CR treatment and lasted until day 10, as indicated by decreased extracellular activity and enzyme release percentage. These findings are surprising to some extent considering that cecropin A is commonly used as a template for peptide molecular hybrids (such as melittin) to enhance the antibacterial activity of AMPs and reduce cell toxicity [6,34]. The effects of treatment with cecropin A were not only the strongest vs. melittin and lactoferrin but also the fastest, as manifested by decreased β-glucosidase activity on day 1. Dosler et al. [35] reported the bactericidal effect of melittin within 2–7 h when tested against Pseudomonas aeruginosa, Escherichia coli and Klebsiella pneumoniae. Ferre et al. [36] showed high synergistic effects of the membrane actions of a cecropin-melittin antimicrobial hybrid peptide BP100, but Leandro et al. [37] did not observe enhanced antimicrobial activity of melittin-phospholipase A2 combinatory use in comparison to melittin alone. In the present study, we compared the action of melittin and cecropin A from insects with that of the well-known antimicrobial protein, namely lactoferrin. Lactoferrin has bacteriostatic properties against *Escherichia coli*, *Staphylococcus aureus, Listeria monocytogenes* and *Bacillus* species and strong action against the formation of biofilms. Some authors reported that infant formula containing LF promoted the growth of beneficial microbiota, such as *Bifidobacteria* and reduced the growth of *Clostridium* in the gut of infants [13].

The activity of β-glucosidase is associated with NSP hydrolysis and degradation of cellulose [38]. Moreover, the changes in β-glucosidase activity are more ambiguous because this hydrolytic activity is responsible for both the generation of toxins and the production of bacterial glucoside derivatives, which are assumed to be responsible for protection against chemically induced cancer [39]. In this regard, β-glucosidase activity is strongly involved in large gut polyphenol metabolism. It is well known that polyphenols are commonly present in plant foods as a bound form, most often conjugated as glycosides, and mainly metabolised by the gut microbiota, resulting in the formation of biologically active aglycones [40]. According to the literature, in addition to a wide range of other species, β-glucosidases are effectively produced by the probiotic bacteria Bifidobacterium and Lactobacillus [41]. However, it has been reported that the number of alkali-tolerant intestinal bacteria (1% of the total microbiota in humans and 0.8% of the microbiota in rats) is characterised by high synthesis of both β-glucosidase and β-glucuronidase. The β-glucosidase and β-glucuronidase activity of these intestinal bacteria is induced by elevating the pH of the medium (the enzyme activity in a medium of pH 7 is 5- to 10-fold higher than that in a medium of pH 6), but the growth is not changed [42]. In our experiment, the dietary addition of 100 mg/kg lactoferrin, melittin or cecropin A caused a significant increase in faecal pH values vs. the control on day 2 (treatment LF), days 5–8 (all three AMPs) and day 10 (treatment CR). An increase in faecal pH accompanied by decreases in bacterial enzymatic activities, including β-glucosidase and β-glucuronidase activities, suggested that all applied AMPs efficiently limit the growth of detrimental bacteria at more alkali pH intestinal values. Interesting and promising results were obtained by Zhai and co-workers [6], who showed selective enrichment of caecal Lactobacillus followed by intraperitoneal injection of 15 mg/kg cecropin A into C57BL/6 mice with DSS-induced IBD. Those authors observed that both cecropin A and the antibiotic gentamicin effectively reduced the number of caecal Bacteroidaceae and Enterobacteriaceae, but the action towards Lactobacillus was entirely different.

The observed faecal pH rise itself was not an unexpected result considering that decreased metabolic activity of intestinal microbiota, especially extracellular enzymatic activity, resulted in lowered SCFA concentrations. The acidity of intestinal contents is determined by various factors, including SCFA and ammonia concentrations, as well as the buffering capacity of the digesta. It has been reported that buffering capacity and SCFA concentrations are variables of paramount importance, whereas ammonia concentration is only slightly positively related to large gut pH values [43,44]. The latter statement about the intraluminal ammonia level and its effect on intestinal pH corroborates our results, as a large decrease in faecal ammonia exerted by LF, MT and CR, e.g., on days 6–8, did not lead to an acidifying effect in the faeces.

## 5. Conclusions

The results suggested that the faecal intensity of microbial fermentation processes in rats was quickly reduced upon dietary addition of the AMPs lactoferrin, melittin or cecropin A at a dosage of 100 mg/kg after two days of treatment, on average. Considering that the rats were subjected to AMPs for 5 days, the strongest suppression effect was observed on the last day of treatment and persisted on days 5–8. The changes caused by the supplemented AMPs were reversible after 15 days, i.e., 10 days after the withdrawal of the AMPs from the diet. While the observed suppression effect on microbial fermentation processes is intended to be maintained, the dietary continuous presence of AMPs ought to be considered as nutritionally advisable, otherwise gut microbiota are likely to return to their pre-supplementation condition. The strongest decrease in bacterial β-glucosidase and β-glucuronidase activities as well as diminished SCFA and ammonia concentrations in the faeces were observed in rats fed a diet supplemented with cecropin A. The mechanism of suppressed microbial fermentation involved a diminished release of enzymes from bacterial cells to the faecal digesta only in the case of cecropin A. In this context our next experiment will be dedicated to very intriguing hypothesis driven by Ramin and Allison [45] that bacteria display three “behaviour” strategies, namely (i) investment in extracellular enzyme production, (ii) investment in high growth rates, or (iii) the struggle for survival with a low potential for enzyme production and growth.

## Figures and Tables

**Table 1:** Composition of control (S) and experimental diets supplemented with lactoferrin (LF), melittin (MT) or cecropin A (CR).

Ingredient (%)	S	LF	MT	CR
Casein ^1^ DL-methionine Choline chloride	20	20	20	20
	0.3	0.3	0.3	0.3
	0.2	0.2	0.2	0.2
Saccharose Cellulose	10	10	10	10
	8.0	8.0	8.0	8.0
Rapeseed oil Rapeseed oil with lactoferrin ^2^ Rapeseed oil with melittin ^3^ Rapeseed oil with cecropin A ^4^	8.0	0	0	0
	0	8.0	0	0
	0	0	8.0	0
	0	0	0	8.0
Cholesterol Mineral mix ^5^	0.3	0.3	0.3	0.3
	3.5	3.5	3.5	3.5
Vitamin mix ^6^	1.0	1.0	1.0	1.0
Maize starch ^7^	48.7	48.7	48.7	48.7

^1^ Casein preparation: crude protein 89.7%, crude fat 0.3%, ash 2.0%, and water 8.0%. ^2^ Lactoferrin, SIGMA (L4765, Poznań, Poland), ≥85% (SDS-PAGE). ^3^ Mellitin, SIGMA (M2272, Poznań, Poland), ≥85% (HPLC). ^4^ cecropin A, SIGMA (C6830, Poznań, Poland), ≥97% (HPLC).^5^ AIN-93G-MX, per kg mix: 357 g calcium carbonate anhydrous (40.04% Ca), 196 g potassium phosphate monobasic (22.76% P, 28.73% K), 70.78 g potassium citrate, tripotassium monohydrate (36.16% K), 74 g sodium chloride (39.34% Na, 60.66% Cl), 46.6 g potassium sulfate (44.87% K, 18.39% S), 24 g magnesium oxide (60.32% Mg), 6.06 g ferric citrate (16.5% Fe), 1.65 g zinc carbonate (52.14% Zn), 1.45 g sodium meta-silicate × 9H2O (9.88% Si), 0.63 g manganous carbonate (47.79% Mn), 0.3 g cupric carbonate (57.47% Cu), 0.275 g chromium potassium sulfate × 12H2O (10.42% Cr), 81.5 mg boric acid (17.5% B), 63.5 mg sodium fluoride (45.24% F), 31.8 mg nickel carbonate (45% Ni), 17.4 mg lithium chloride (16.38% Li), 10.25 mg sodium selenate anhydrous (41.79% Se), 10 mg potassium iodate (59.3% I), 7.95 mg ammonium paramolybdate × 4H2O (54.34% Mo), 6.6 mg ammonium vanadate (43.55% V), 221.026 g powdered sucrose. ^6^ AIN-93G-VM, g/kg mix: 3.0 nicotinic acid, 1.6 Ca pantothenate, 0.7 pyridoxine-HCl, 0.6 thiamin-HCl, 0.6 riboflavin, 0.2 folic acid, 0.02 biotin, 2.5 vitamin B-12 (cyanocobalamin, 0.1% in mannitol), 15.0 vitamin E (all-rac-α-tocopheryl acetate, 500 IU/g), 0.8 vitamin A (all-trans-retinyl palmitate, 500,000 IU/g), 0.25 vitamin D-3 (cholecalciferol, 400,000 IU/g), 0.075 vitamin K-1 (phylloquinone), 974.655 powdered sucrose. ^7^ Maize starch preparation: crude protein 0.6%, crude fat 0.9%, ash 0.2%, total dietary fibre 0%, and water 8.8%.

## Data Availability

Data is available from the corresponding author upon reasonable request.
